# Predicting Microbiome Function Across Space Is Confounded by Strain-Level Differences and Functional Redundancy Across Taxa

**DOI:** 10.3389/fmicb.2020.00101

**Published:** 2020-02-07

**Authors:** Elle M. Barnes, Erin L. Carter, J. D. Lewis

**Affiliations:** ^1^Department of Biological Sciences, Louis Calder Center – Biological Field Station, Fordham University, Armonk, NY, United States; ^2^Department of Biological Sciences and Center for Urban Ecology, Fordham University, Bronx, NY, United States

**Keywords:** *Batrachochytrium dendrobatidis*, microbiome, disease, urbanization, functional redundancy, spatial variation

## Abstract

Variation in the microbiome among individual organisms may play a critical role in the relative susceptibility of those organisms to infection, disease, and death. However, predicting microbiome function is difficult because of spatial and temporal variation in microbial diversity, and taxonomic diversity is not predictive of microbiome functional diversity. Addressing this issue may be particularly important when addressing pandemic diseases, such as the global amphibian die-off associated with *Bd*. Some of the most important factors in probiotic development for disease treatment are whether bacteria with desired function can be found on native amphibians in the local environment. To address this issue, we isolated, sequenced, and assayed the cutaneous bacterial communities of *Plethodon cinereus* along a gradient of land use change. Our results suggest that cutaneous community composition, but not overall diversity, change with changes in land use, but this does not correspond to significant change in *Bd*-inhibitory function. We found that *Bd*-inhibition is a functionally redundant trait, but that level of inhibition varies over phylogenetic, spatial, and temporal scales. This research provides further evidence for the importance of continued examination of amphibian microbial communities across environmental gradients, including biotic and abiotic interactions, when considering disease dynamics.

## Introduction

The diversity of microbes on an individual has been implicated in the health and resilience of organisms in many taxa, from plants (Mendes et al., 2013) to animals (Yildirim et al., 2010; Sanchez et al., 2012; Bahrndorff et al., 2016), including humans (Cho and Blaser, 2012; The Human Microbiome Project Consortium et al., 2012b). For instance, the presence of infection or disease is driven by the diversity of, and interactions between, pathogenic and non-pathogenic microbiota and the host’s immune system (Blaser, 2006; Benson et al., 2009, 2010; Blaser and Falkow, 2009; Maldonado-Contreras et al., 2011; Feng et al., 2017). Yet, the taxonomic diversity of microbes on an organism is an imperfect predictor of functional diversity (Gilbert et al., 2010; Delgado-Baquerizo et al., 2016; Widder et al., 2016). Predicting community function is further confounded because microbiomes vary extensively over space and time. Hence, predicting the role of microbiota in regulating infection and disease requires a better understanding of spatial and temporal variation in both taxonomic and functional diversity (The Human Microbiome Project Consortium et al., 2012a; Lloyd-Price et al., 2017; Kolde et al., 2018).

Bacterial communities may exhibit substantial biogeographic variation at both local (Costello et al., 2009; Bakker et al., 2014; Zhang et al., 2014) and continental scales (Fierer and Jackson, 2006; Meyer et al., 2018). Further, genetic divergence among populations of bacteria is common, due to the limited ability of bacteria to cross geographic barriers, their short generation times, and high population densities (Martiny et al., 2006; Taylor et al., 2006; Kuehne et al., 2007; Krause and Whitaker, 2015). So, while some genera may appear to be cosmopolitan (Fenchel et al., 1997), morphological and functional differences can arise from geographic divergence, creating distinct ecotypes or strains (Cohan, 2002; Bissett et al., 2010; Booth et al., 2016).

An emerging approach to study how microbial communities respond to environmental differences at both local and regional spatial scales is analyzing the effects of land use change. Habitat destruction, intensification of agriculture, and urbanization all alter edaphic properties, with correlated effects on microbial community composition (Zhao and Guo, 2010; Suleiman et al., 2013; Hawkes and Keitt, 2015; Plassart et al., 2019). In particular, urbanization may markedly alter the physicochemical properties of soil, leading to changes in the abundance and taxa of soil microbes (Zhao and Guo, 2010; Xu et al., 2014; Reese et al., 2016). Studies of the effects of land use change, and spatial variation in microbiota more generally, have rapidly increased in prevalence due to improvements in sequencing technologies (Caporaso et al., 2012; Lal and Seshasayee, 2014; Thompson et al., 2017; Averill et al., 2019); of critical importance now is understanding how these differences relate to community function (Robinson et al., 2010; Belzer and de Vos, 2012; Durrant and Bhatt, 2019).

Studies of genes linked to key environmental processes have related disturbances to changes in both microbial taxonomic and functional diversity (Bissett et al., 2011; Paula et al., 2014; Plassart et al., 2019), yet microbiomes usually are classified solely using the 16S rRNA gene. As others note, this gene alone is not a reliable indicator of the functional ability of a microbiome (Mollet et al., 1997; Fukushima et al., 2002; Hilario et al., 2004; Janda and Abbott, 2007; Becker et al., 2015). This weakness partly is because genomic diversity is high within microbial species, and adaptation to the local habitat can change the gene pool, creating sequence-discrete populations (Philippot et al., 2010; Smillie et al., 2011; Mell and Redfield, 2014; Shapiro and Polz, 2014; Becker et al., 2015; Gómez et al., 2016; Garcia et al., 2018; Ellegaard and Engel, 2019). Thus, 16S data may underestimate functional ability of microbiomes, due to strain-level adaptations to local conditions, or overestimate functional ability, due to functional redundancy across taxa (Delgado-Baquerizo et al., 2016; Mori et al., 2016; Vieira-Silva et al., 2016). As a result, understanding the complex interactions that shape microbial community assembly and structure requires complementing taxonomic analyses with assessments of functional differences within species and across environments (Burke et al., 2011; Jiang et al., 2019).

The current global amphibian decline is a critical system for assessing associations between microbial diversity and disease within a conservation context (Wake and Vredenburg, 2008; Ceballos et al., 2015; Bower et al., 2017). The decline is driven by the combined effects of habitat degradation and the fungal disease, *Batrachochytrium dendrobatidis* (*Bd*) (Collins and Storfer, 2003; Stuart et al., 2004). Amphibian infection by *Bd*, however, is blocked by some species of cutaneous bacteria, such as *Janthinobacterium lividum*, which produce antifungal metabolites and compete for space and resources with each other and the fungus (Longcore et al., 1999; Becker and Harris, 2010; Familiar López et al., 2017; Bates et al., 2018). However, it is still unknown in a variety of systems if these functional defense mechanisms, which many times are assumed to remain consistent with taxonomic identity, actually differ over space and time (Daskin et al., 2014; Jia and Whalen, 2019).

To address this issue, we examined bacterial diversity and function of the salamander microbiome across a 64 km gradient of land use change in the New York metropolitan area (NY, USA). We hypothesized that both composition and diversity of the cutaneous community would differ with urbanization: urban communities comprised of mainly cosmopolitan species, exurban communities of locally unique species, and suburban a mix of both making it the most diverse. In addition, we hypothesized that *Bd*-inhibitory ability would be a widespread and redundant trait but would increase with urbanization. This is because bacteria regularly come in contact with and compete with other fungi, not only *Bd*. While *Bd* prevalence has been suggested to be lower in urban areas (Becker and Zamudio, 2011), large urban areas such as New York are known centers for urban invaders and major points of entry for amphibians and *Bd* in the wildlife pet trade. Additionally, New York has the optimal climate for *Bd* persistence even if hosts are unavailable during certain seasons (Schloegel et al., 2009). Given that urban bacteria exist in areas with adverse environmental conditions and an increase in invaders, higher *Bd*-resistance could be an indirect benefit of their general response to these stressors. Lastly, we also predicted inhibition to vary among isolates of the same species based on location and season due to limitations in dispersal and potential local adaptations. The results of this study further uncover inhibitory bacteria in novel environments, such as urban areas, and aid in the discovery of potential native probiotics for other at-risk amphibian species in this species-rich region.

## Methods

### Study Species

A terrestrial species of salamander, the eastern redback salamander (*Plethodon cinereus*), was sampled at each of the nine study sites (*n* = 65 across all sites). We chose eastern redback salamanders for this study because they are locally abundant and are one of the most commonly used amphibians for studies on *Bd*-resistance in the United States. Chytridiomycosis has not been demonstrated to affect *P. cinereus* in nature, but chytridiomycosis symptoms can be induced in artificially infected salamanders by removing or augmenting their microbiome (Harris et al., 2009a,b; Becker and Harris, 2010). Thus, because this salamander can be infected by *Bd* but is not susceptible to it, studying its microbiome may provide the key to finding anti-fungal bacteria to use on vulnerable individuals. Permission to sample *P. cinereus* was obtained through the New York State Department of Environmental Conservation (permit no. 1159), the New York City Department of Parks and Recreation (permits 2016-2018), and the New York State Office of Parks, Recreation, and Historic Preservation (permit no. 2016-MP-008) and the Fordham University IACUC #JL-17-01.

### Study Sites

**Figure 1:**
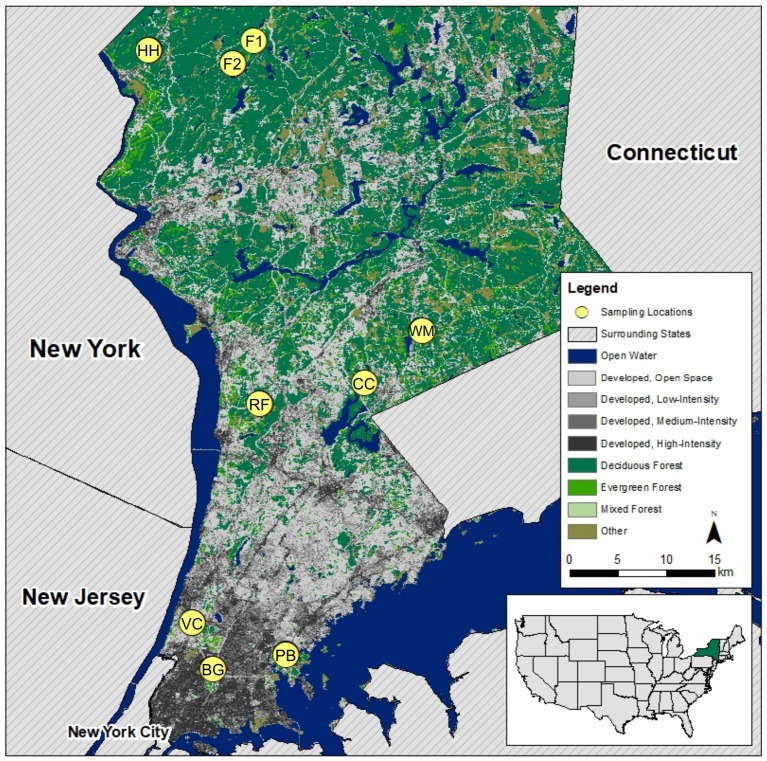
Map of sampling locations along urban-to-exurban gradient showing developed land cover (from low to high intensity) in gray and forest in green adapted from the National Land Cover Database (NLCD) (2011) United States land cover dataset. Site code used throughout the manuscript is shown within the marker to identify each sampling location. Map in bottom-right corner locates New York State within the USA. Sites were classified as urban, suburban, or exurban based upon each site’s distance from Central Park in New York City, human population density, and GIS landcover analysis of percent-developed land within 5 miles of each study site.

This research took place in parks and preserves in New York City and the two counties north of the city (Westchester and Putnam, NY, USA). We sampled from a total of nine study sites (Figure 1), three sites in each of the following categories: urban (Van Cortlandt Park, the New York Botanical Garden, and Pelham Bay Park), suburban (Rockefeller State Park Preserve, Fordham University’s Louis Calder Center, and Westmoreland Sanctuary), and exurban (two sites within Clarence Fahnestock State Park and one within Hudson Highlands State Park Preserve). Study site classification was based upon each site’s distance from Central Park in New York City, human population density, and GIS landcover analysis of percent-developed land within 5 miles of each study site (urban: >80%, suburban: 20–80%, exurban: <20%). All sites were located in mixed deciduous forests in New York State east of the Hudson River.

### Sampling

Samples were collected in Fall 2016 (September through October) and Spring 2017 (April through May), except for 10 samples taken from salamanders at Fahnestock and Hudson Highlands State Parks in Spring 2018. Salamanders were captured and handled using sterile gloves, which were changed between each individual. A non-invasive sampling method of bacterial swabbing was used based on Boyle et al. (2004). In brief, each salamander was rinsed twice with 25 ml of sterile water and swabbed using a sterile cotton swab on their ventral, right, and left sides (Harris et al., 2006). Swabs were immediately streaked onto LB agar (5 g NaCl, 5 g tryptone, 2.5 g yeast extract, 7.5 g agar, 200 μl 2.5 N NaOH, and 500 ml H_2_O) and were incubated at ambient temperature for 72 h. We chose to use LB agar to provide a high nutrient alternative to R2A, which is commonly used in amphibian microbiome studies as the nutrient content of salamander skin is still undetermined. Instead, we focused on swabbing a higher number of individuals across our sites to maximize culture diversity regardless of media (Medina et al., 2017b).

### Identification of Bacterial Isolates

For each individual, we isolated bacteria based on morphotype (colony shape, elevation, margin, surface, and color) to pure cultures on LB agar. DNA from these cultures was extracted using the Qiagen DNeasy Blood and Tissue kit (Germantown, MD, USA) following manufacturer’s protocols, with the addition prior to purification of lysozyme incubation for Gram-positive bacteria. DNA was amplified with the 515F (5′-GTGYCAGCMGCCGCGGTAA-3′) and 806R (5′-GGACTACNVGGGTWTCTAAT-3′) primer pair, which targets the V4 region of the 16S rRNA gene, in 25 μl reactions: 12.5 μl GoTaq Green PCR Master Mix (2X), 1 μl forward primer, 1 μl reverse primer, 2 μl DNA, and 8.5 μl nuclease-free water. PCR protocols included: 95°C for 5 min, followed by 95°C for 1 min, 55°C for 1 min, and 72°C for 1 min for 30 cycles, and 72°C for 5 min. This primer pair was chosen as it targets a small region commonly sequenced by high-throughput 16S rRNA sequencing, allowing our results to be easily compared to results from other studies. Amplicon lengths were assessed using gel electrophoresis (1% agarose), and samples yielding amplicons of ~250 bp were then sequenced using Sanger sequencing at Macrogen (New York, NY, USA).

Forward and reverse amplicon sequences were aligned to obtain a consensus sequence for each isolate in Geneious R11.1 (Biomatters Inc., Auckland 1010, New Zealand). Fourteen sequences were discarded due to poor read quality. The remaining 204 sequences were then run in the EZBioCloud database, a curated and integrated database of all 16S rRNA bacterial sequences from NCBI, to assign taxonomy (Yoon et al., 2017). OTU identification was assigned based on the closest reference sequence match with a taxonomic classification if sequence similarity was >97%. In instances where sequence similarity was >97% match to two or more OTUs, samples were classified based on highest percent query cover and completion of read. Additionally, 16S rRNA gene trees for isolates within each level of urbanization were built with MrBayes v. 3.2.6 (Huelsenbeck and Ronquist, 2001). Our runs consisted of four simultaneous Markov chains, each with 1,000,000 generations, a subsampling frequency of 200, and a burn-in fraction of 0.15. Trees were then visualized and adapted in FigTree v. 1.4.3 (Rambaut, 2012).

### Characterization of Anti-*Bd* Ability

All bacterial isolates (including multiple isolates of the same OTU) were challenged with *Bd* using the 96-well cell-free supernatant (CFS) method developed by Bell et al. (2013). Samples of *Bd* strain JEL423, a hypervirulent strain from the global panzootic lineage *Bd*GPL (Farrer et al., 2011; DiRenzo et al., 2014), were obtained from Dr. Joyce Longcore (University of Maine). Each isolate was diluted to 1:10^4^ cfu and plated on LB agar plates until colonies formed, after which 1 cm^2^ of agar with colonies was excised and placed in 7 ml of 1% tryptone broth and allowed to grow at 23°C for 48 h. To obtain *Bd* used in each microcosm, 1 ml of *Bd* culture was plated on 1% tryptone plates and grown at 23°C for 4 days, after which 25 ml of 1% tryptone broth was inoculated with decanted zoospores. This *Bd* liquid culture was then grown at 23°C for 48 h.

Each isolate was co-cultured with 2 × 10^6^ zoospores *Bd* (100 μl of each) in 800 μl of 1% tryptone, a minimal-nutrient media in which *Bd* often is grown (Longcore et al., 1999; Piotrowski et al., 2004), for 72 h at 23°C. Cultures were checked for turbidity at OD600 to confirm that they were in late log to early stationary phase and then centrifuged for 5 min at 10,000*g*. Cultures that did not reach these phases (<5% of isolates) were incubated for an additional 24 h. The supernatant was then filtered through a sterile, 0.22-μm cellulose acetate syringe filter (VWR, Radnor, PA, USA) to obtain CFS. CFS was then assayed with *Bd* (50 μl CFS: 50 μl *Bd*-zoospore suspension) in triplicate in a 96-well plate for 10 days at 23°C. The *Bd*-zoospore suspension consisted of growing *Bd* on a 1% tryptone agar plate for 4 days, flooding the plate with 3 ml 1% tryptone, and then filtering this suspension through a 20-μm filter. Zoospore concentration was estimated at 2 × 10^6^ zoospores per ml using an INCYTO C-Chip™ hemocytometer (SKC Inc., Covington, GA, USA). Both positive (50 μl *Bd* CFS: 50 μl *Bd*-zoospore suspension) and negative controls (50 μl *Bd* CFS: 50 μl heat-killed *Bd*-zoospore suspension) were included to control for potential variation in *Bd* zoospore concentrations, *Bd*-produced metabolites, and *Bd* growth when alone. Optical density was measured at 0, 4, 7, and 10 days using an Infinite 200 Pro microplate reader (Tecan Trading AG, Männedorf, Switzerland).

To calculate *Bd* inhibition, absorbance readings were transformed using the equation: ln[OD/(1-OD)] following Becker et al. (2015). A linear regression was run to determine the growth rate of *Bd* in the presence of CFS from each isolate. The average slope of each triplicate was then divided by the average growth rate of the positive control, and this value was subtracted from one. This calculated value represented percent *Bd* inhibition, and bacterial OTUs were then classified by taking the mean percentage inhibition of multiple isolates of the same OTU.

### Statistical Analysis

Alpha diversity was calculated using the Shannon index due to its reduced sensitivity to sampling depth differences, as compared to Chao-1 (Haegeman et al., 2013; Preheim et al., 2013). Chi-square test of independence was used to account for the relationship between year and site in our sampling. Beta diversity between sites was measured by generating a Bray-Curtis distance matrix on OTU-level presence-absence data (Legendre and Legendre, 1998) and conducting a permutational multivariate analysis of variance (PERMANOVA) with each site nested by level of urbanization. These data were then used for ordination by nonmetric multidimensional scaling (NMDS) with 1,000 randomized runs. Pairwise analyses were conducted using the package *pairwiseAdonis* (Martinez, 2019). All analyses were conducted in *vegan* v. 2.5-2 in R 3.6.0 (Oksanen et al., 2013).

We examined the distribution of isolate inhibition using Hartigan’s dip test (Hartigan and Hartigan, 1985) with the *diptest* package (Maechler and Ringach, 2013) to test for multimodality. The distribution was then visualized using the EM algorithm in the *mixtools* package (Benaglia et al., 2009), and the mean and standard deviation of each mode was calculated. We compared each isolate’s percent inhibition with each plate’s *Bd* control using a Mann-Whitney U-test to classify *Bd*-inhibitory ability. Differences among bacterial OTUs, genera, phyla, site, and level of urbanization were tested with a Kruskal-Wallis test followed by Dunn *post-hoc* test. Differences in level of inhibition between the Spring and Fall seasons were tested with a Mann-Whitney U-test.

## Results

### Community Diversity and Composition

**Figure 2:**
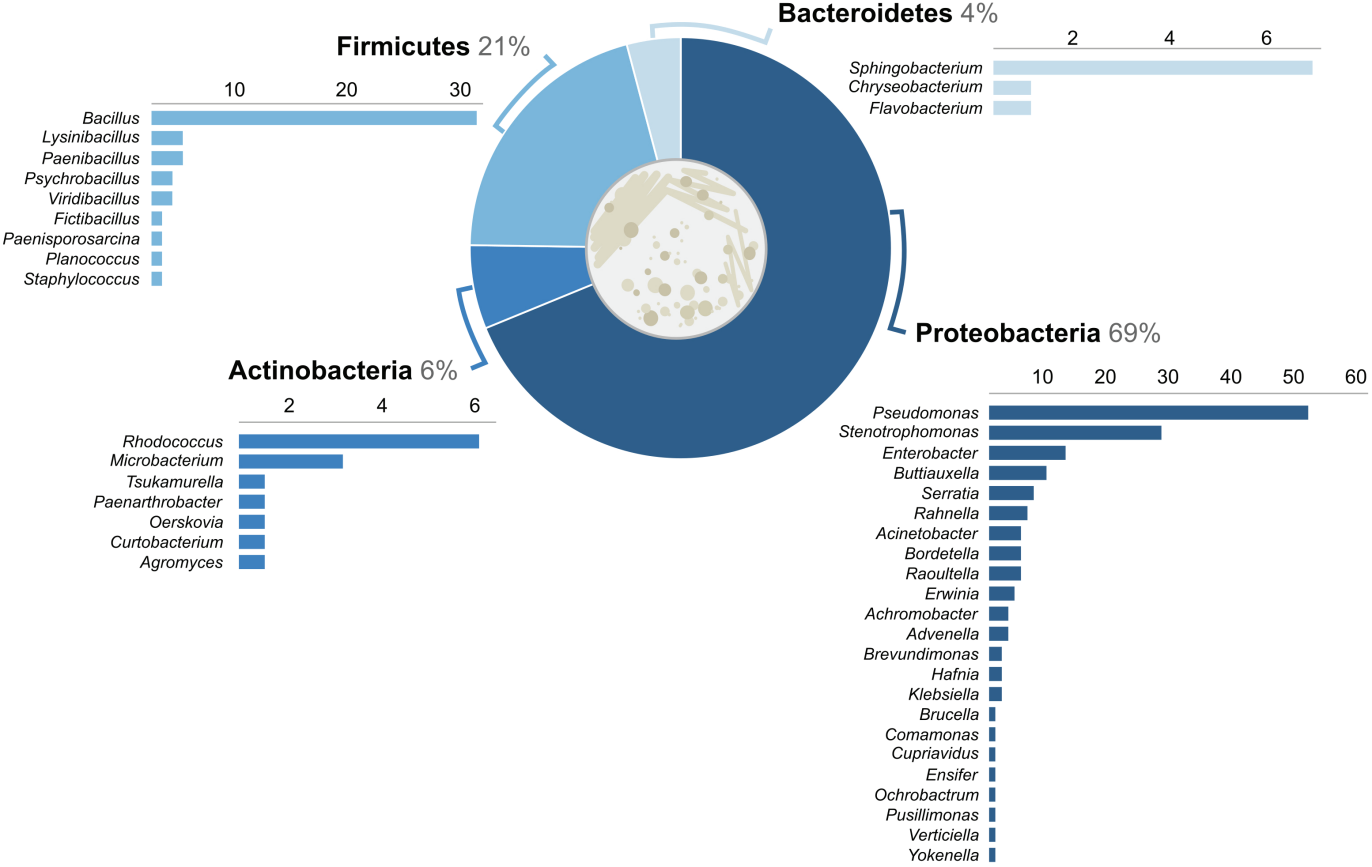
Percent of bacterial isolates (*n* = 204) across the gradient by phylum. Number of isolates identified within genera is displayed below each phylum.

One of our primary objectives was to characterize the diversity of bacteria on salamanders from a large urban area in the northeastern USA. From 69 salamanders, a total of 218 isolates were cultured, 204 of which could be identified as OTUs (Table 1). Four phyla were identified: Proteobacteria (69%), Firmicutes (21%), Actinobacteria (6%), and Bacteroidetes (4%) (Figure 2). Out of 42 total genera, the three most common genera were *Pseudomonas* (43 isolates), *Bacillus* (28 isolates), and *Stenotrophomonas* (26 isolates). The majority of other genera were represented by less than 10 isolates each. While no one bacterial OTU was found at all nine sites, *Stenotrophomonas rhizophila* was found across all three levels of urbanization and at seven out of nine sites.

**Table 1:** Summary of salamanders sampled across nine sites in three levels of urbanization.

Level urbanization	Site	No. Indls sampled (*n* = 69)	No. isolates (*n* = 218)	No. Genera; Most common	No. OTUs; Most common	Diversity (H)	
Urban	Van Cortlandt Park (VC)	7	28	12; *Pseudomonas* (PS)	19; *B. gaemokensis* (BAGA)	2.82	Total: 3.44
	New York Botanical Garden (BG)	8	20	11; *Bacillus* (BA)	16; *P. helmanticensis* (PSHE)	2.69	
	Pelham Bay Park (PB)	9	29	13; *Bacillus* (BA)	21; *S. rhizophila* (STRH) *B. wiedmannii* (BAWI)	2.95	
Suburban	Rockefeller State Park Preserve (RF)	11	29	12; *Pseudomonas* (PS)	17; *E. cloacae* (ENCL) *B. wiedmannii* (BAWI) *A. modestus* (AIMO)	2.65	Total: 3.48
	Louis Calder Center (CC)	5	16	9; *Pseudomonas* (PS)	13; *S. myotis* (SEMY)	2.48	
	Westmoreland Sanctuary (WM)	10	27	15; *Stenotrophomonas* (ST)	22; *E. cloacae* (ENCL)	3.02	
Exurban	Fahnestock State Park 1 (F1)	13	42	15; *Pseudomonas* (PS)	24; *R. inusitata* (RAIN)	3.01	Total: 3.29
	Fahnestock State Park 2 (F2)	4	16	6; *Buttiauxella* (BU)	6; *B. gaviniae* (BUGA)	1.68	
	Hudson Highlands (HH)	2	11	9; *Bacillus* (BA) *Pseudomonas* (PS)	10; *B. gaemokensis* (BAGA)	2.27	

*Site codes and OTU abbreviations (genus and species) are in parentheses. Diversity is shown for each site and for level of urbanization and was calculated using the Shannon Index*.

OTU richness and abundance varied by site (Table 1) and a chi-square test of independence showed that both year and site were correlated with regard to OTU richness (*X*^2^ = 137.4, *p* < 0.05). While there was no trend of richness with land use type (*p* = 0.73), only 11 genera were found at all three levels of urbanization. These include: *Pseudomonas, Bacillus, Stenotrophomonas, Sphingobacterium, Serratia, Rhodococcus, Microbacterium, Lysinibacillus, Enterobacter, Buttiauxella,* and *Bordetella*. The remaining 31 genera were unique to certain levels of urbanization, with 24 genera (42% of total genera) unique to a single site. Interestingly, the most common OTU at each site often belonged to a different genus than the genus found to be most common at that site (Table 1).

**Figure 3:**
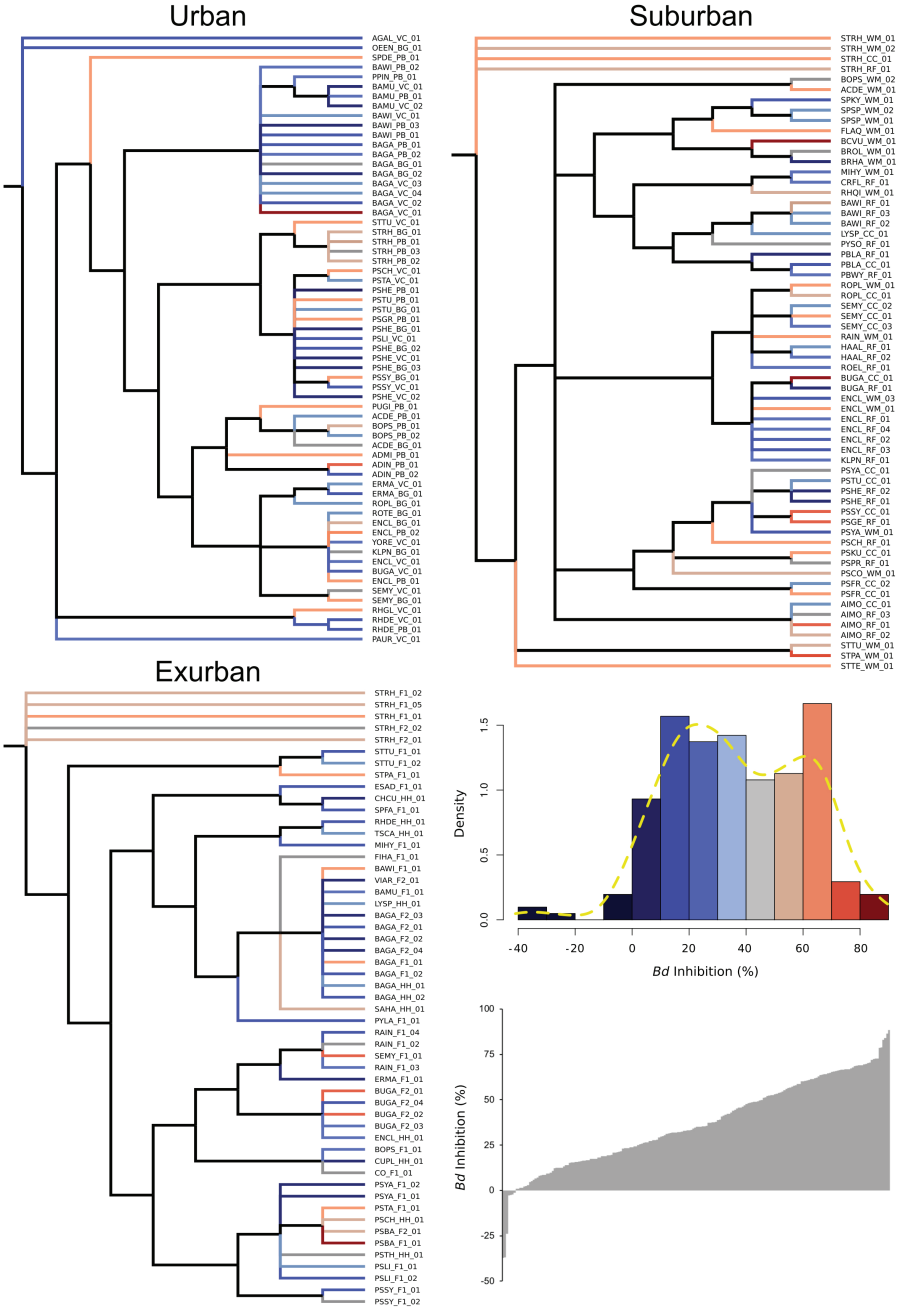
Salamander skin bacterial OTUs (*n* = 204) at each level of urbanization displayed as phylogenies. OTUs are identified by the first two letters of the genera and species names (from the 16S rRNA sequence) followed by the site code and isolate number. Color of each isolate’s branch on phylogenetic tree corresponds to *Bd*-inhibitory ability. *Bd*-inhibitory ability is divided into intervals of 10% and color is denoted as displayed in the density histogram. Cooler colors indicate low *Bd*-inhibition, whereas warmer colors indicate high *Bd*-inhibition. Dashed yellow line in the density histogram shows a bimodal distribution of *Bd*-inhibition (*p* = 0.030).

**Figure 4:**
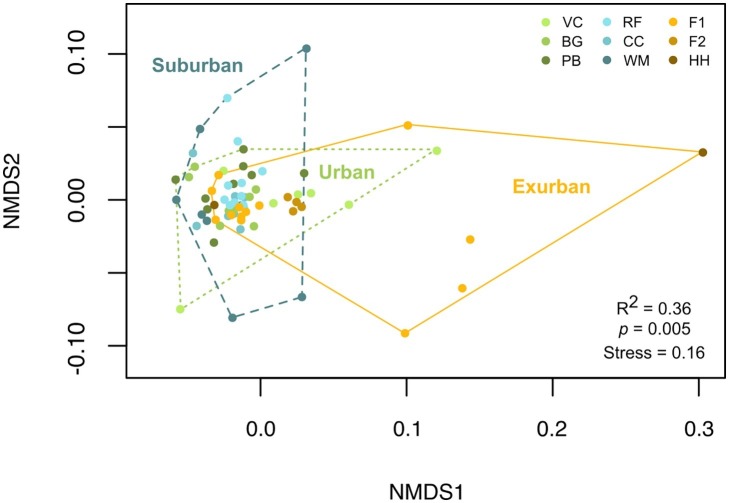
NMDS of beta diversity by individual salamander (*n* = 69) blocked by level of urbanization. Beta diversity was measured by calculating a Bray-Curtis dissimilarity matrix of OTU-level presence-absence culture data. Color denotes sampling location. PERMANOVA of beta diversity by level of urbanization indicates a significant difference between suburban and exurban skin bacterial communities (*R*^2^ = 0.375, *p* = 0.005).

While urban sites had the greatest number of individual isolates compared to suburban and exurban sites, suburban sites had the largest overall Shannon’s diversity score, 3.48 (*p* = 0.56, Table 1). However, high OTU richness did not correlate to phylogenetic diversity (Figure 3), as many of the isolates were closely related and came from a small number of genera. An NMDS of beta diversity indicated that urban, suburban, and exurban bacterial communities grouped by level of urbanization (*R*^2^ = 0.36, *p* = 0.005; Figure 4). Urban and suburban communities overlapped to the left of the plot, but urban communities distributed along the first axis while suburban communities distributed along the second axis. Exurban sites separated to the right of the ordination plot, with some overlap with urban communities and a pairwise PERMANOVA revealed a significant difference with suburban communities (*R*^2^ = 0.15, *p* = 0.003).

### Functional Redundancy

To assess whether *Bd*-inhibitory ability was multimodally distributed in our sample of salamanders, we used Hartigan’s dip test. We observed that *Bd*-inhibitory ability was bimodal (*p* = 0.030), exhibiting at least two groups of isolates: the first with a mean of 25.9% (SD = 18.5%), and the second with a mean of 63.9% *Bd*-inhibition (SD = 9.5%) relative to the control (Figure 3). Isolates within 1.5 SD of the second mean (all isolates above 49.6% *Bd* inhibition) were considered inhibitory—32% of all isolates. Isolates above the mean of the first mode but below 1.5 SD of the second mean (i.e., isolates between 25.9 and 49.6% *Bd* inhibition) were considered mildly inhibitory—30% of isolates, while isolates below this group but within 1.5 SD of the mean of the first mode were considered to have no effect on *Bd*-growth—34% of isolates. Lastly, the seven isolates (3%) that fell below this threshold were considered facilitative: increased *Bd*-growth relative to the control.

**Figure 5:**
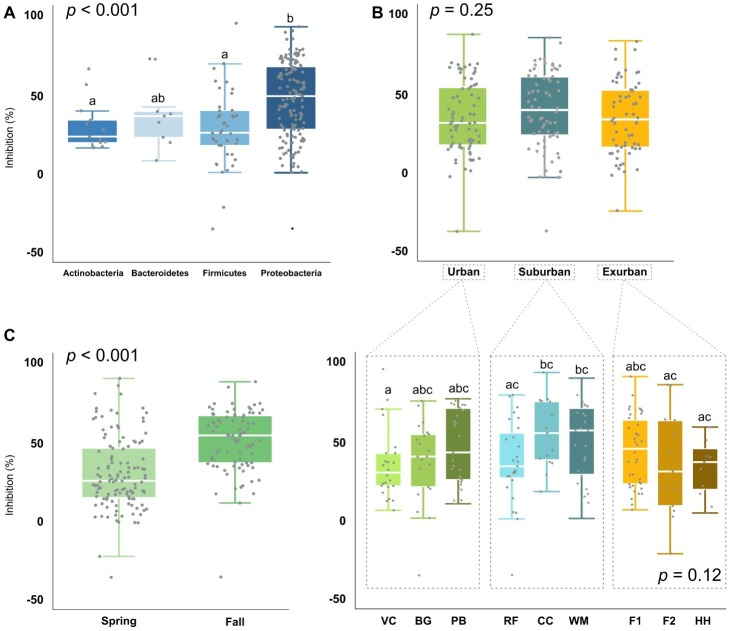
*Bd*-inhibition of isolates (*n* = 204) by **(A)** phylum (*p* < 0.001), **(B)** level of urbanization (*p* = 0.252) and site [*p* = 0.117, but significant (*p* < 0.05) pairwise site effects], and **(C)** season (*p* < 0.001).

To assess whether *Bd*-inhibitory function was phylogenetically unique vs. redundant across those groups, we analyzed isolate *Bd* inhibition by phylum, genus, and OTU. When analyzed by phylum, Proteobacteria were on average 68% more inhibitory than Actinobacteria (*p* = 0.004) and Firmicutes (*p* < 0.001) and 26% more inhibitory than Bacteroidetes (*X*^2^ = 22.1, Figure 5A). At the genus and OTU level, isolates exhibited a large range of inhibitory ability, with few distinct phylogenetic patterns (Figure 3). In some extreme cases, isolates within the same genus, such as *Bacillus and Pseudomonas* (identified as “BA” and “PS” on the branch tips of the phylogenetic trees), were identified as inhibitory, while others were facilitative to *Bd* growth (*p* < 0.05). When we compared a subset of the most common bacterial genera (*n* ≥ 5 isolates), we found differences between genera (*p* = 0.012), driven by two genera: *Bacillus* (non-inhibitory, mean = 25%) and *Stenotrophomonas* (inhibitory, mean = 54%) (Table 2).

**Table 2:** Anti-*Bd* activity of bacterial genera with greater than five isolates.

		Inhibition of Bd (%)			
Genus	No. inhibitory isolates/total	Mean	SD	Range	Mean by level of urbanization
*Pseudomonas*^†^^,^^§^	16/43	37.17	25.6	−2.7 to 83.9	*U* = 29.6 *S* = 43.2 *E* = 40.9
*Bacillus*^§^	4/28	25.0	24.1	−37.1 to 88.5	*U* = 23.1 *S* = 39.9 *E* = 23.5
*Stenotrophomonas*^†^	19/26	53.8	11.8	18.2–72.6	*U* = 51.7 *S* = 60.4 *E* = 50.3
*Enterobacter*^§^	4/12	34.9	22.4	10.9–69.1	*U* = 52.1 *S* = 26.1 *E* = 27.7
*Buttiauxella*^†^	4/8	47.8	32.0	9.8–86.4	*U* = 43.6 *S* = 48.1 *E* = 49.8
*Serratia*^†^	3/8	48.1	18.4	24.2–72.5	*U* = 50.0 *S* = 42.9 *E* = 53.0
*Sphingobacterium*^§^	1/7	32.7	16.7	15.7–66.8	*U* = 47.4 *S* = 28.9 *E* = 23.7
*Rhodococcus*^§^	2/5	30.7	23.2	12.5–60.6	*U* = 29.9 *S* = 51.0 *E* = 12.5
*Rahnella*	2/5	42.7	22.8	16.5–66.8	*S* = 66.8 *E* = 36.7
*Acinetobacter*	2/5	34.0	42.4	−36.9 to 72.7	*S* = 34.0
*Bordetella*	2/5	44.2	14.8	24.3–61.9	*U* = 46.5 *S* = 51.8 *E* = 24.3
*Raoultella*	2/5	41.9	17.5	20.8–66.1	*U* = 35.2 *S* = 46.4

*Number of isolates determined to be greater than 49.6% *Bd*-inhibitory are given out of total number of isolates within each genus. Mean level of *Bd*-inhibition displayed by genus and level of urbanization (*U* = urban, *S* = suburban, *E* = exurban)*.

† *Denotes significant difference (*p* < 0.05) from *Bacillus* (non-inhibitory)*.

§ *Denotes significant difference from *Stenotrophomonas* (inhibitory)*.

### Spatial and Temporal Variation Within Genera and Species

We further analyzed *Bd*-inhibitory function by level of urbanization, site, and season. Overall, suburban sites appeared to have the largest proportion of inhibitory isolates with 41%, followed by exurban (34%) and urban (30%). Similarly, individual salamanders also varied in the number of *Bd* inhibitory isolates cultured from their skin. Depending on the site, 17–50% of isolates per individual were >49.5% inhibitory, with salamanders from suburban sites having on average 15% more inhibitory isolates than individuals from urban or exurban sites. However, the effect of urbanization was not statistically significant (*X*^2^ = 2.8, *p* = 0.25, Figure 5B)—so, while there appeared to be fairly large relative differences in the proportion of inhibitory isolates among levels of urbanization, with suburban communities 10–60% more inhibitory than urban and exurban communities (*X*^2^ = 2.5, *p* = 0.28), there was a lot of variation within genera even at the site level.

In addition, *Pseudomonas* and *Bacillus*, which make up a large proportion (35% of all isolates) of the cultured bacterial community, exhibited substantial variation in *Bd* inhibition, with differences by both site (*p* < 0.05) and season (*Pseudomonas*: *p* < 0.001, *Bacillus*: *p* = 0.022). In *Bacillus*, differences were even observed among isolates at the individual site level, such as *B. gaemokensis* between VC and F2 (*p* = 0.021), and *B. wiedmannii* between PB and F2 (*p* = 0.021). In contrast, *Stenotrophomonas* was consistently inhibitory, exhibiting no clear variation in inhibition by level of urbanization, site, season, or OTU. When analyzed by season, bacteria isolated in the fall season were 72% more inhibitory than bacteria in the spring (*W* = 2291.5, *p* < 0.001; Figure 5C). This same trend still held when a subset of the bacteria, which were found at the same site in both seasons, were compared (*W* = 437, *p* = 0.002). When we compared *Bd* inhibition by year, we found no significant difference between both the 2017 and 2018 spring seasons (*X*^2^ = 43.5, *p* = 0.40).

## Discussion

Studies on the distribution of bacteria and the variability of their function are limited (Kueneman et al., 2014; Becker et al., 2015; Bletz et al., 2017; Jiménez and Sommer, 2017; Kruger, 2019) but are increasingly relevant to the amphibian microbiome as changes in microbial community structure have been hypothesized to result in changes in anti-fungal function. In line with these studies, our results suggest that predicting microbiome function across space and time is difficult due to strain-level differences and functional redundancy across taxa. This knowledge is foundational to understanding the dynamic nature of host-associated microbiomes and their function against pathogens, especially given the urgent need to conserve and protect vulnerable taxa in environments around the globe.

A key constraint to understand the distribution and variability of the amphibian microbiome is the limited number of studies in certain geographic areas, as indicated by the studies compiled in the Antifungal Isolates Database (Woodhams et al., 2015). These areas notably include the Northeastern United States, which has comparatively high salamander diversity and urban land use (~11%; Nickerson et al., 2011). Thus, we chose to sample *Plethodon cinereus* along a gradient of land use in the New York metropolitan area. We acknowledge that our sampling design and site selection may result in overlapping effects of land use and site microenvironment. However, this study design ultimately enabled us to better explore how environmental changes over even small geographic distances in these regions can influence variability of host microbial community diversity, composition, and function.

### Community Diversity and Composition

Contrary to our hypothesis, Shannon’s diversity was similar regardless of site or land use. This pattern is in contrast to other studies on the effects of urbanization on plant and animal biodiversity, which suggest that native species richness tends to be reduced in areas with extreme urbanization (Shochat et al., 2006; Faeth et al., 2011; Laforest-Lapointe et al., 2017; Yan et al., 2019). Additionally, the presence of three cosmopolitan genera (*Pseudomonas*, *Bacillus*, and *Stenotrophomonas*) across the entire gradient, representing nearly half (48%) of all isolates, and our observation that urban sites had the highest OTU richness, contrasts with the idea that urbanization generally drives homogenization of taxa in these communities at least for bacteria (McKinney, 2006). However, we did find that site and year were correlated with regard to richness likely due to poor sampling conditions at Fahnestock and Hudson Highlands in 2017 leading us to also sample only those sites in Spring 2018. Despite this, we found no significant difference in *Bd* inhibition with year regardless of site.

While our use of culturing to examine overall taxonomic diversity is known to miss a proportion of taxa, it allowed us to assess diversity of many actives, and likely functionally relevant, bacteria on salamanders at these locations. However, we do acknowledge that the results of culturing alone are sensitive to the growth media chosen and does not enable us to distinguish bacterial relative abundances as they occur on salamander skin. Our results suggest that while diversity does not differ with urbanization, taxonomic composition does. This pattern has been seen repeatedly in other studies on microbial communities, which suggest that urbanization likely impacts relative abundance of taxa in local communities rather than overall diversity (Cousins et al., 2003; Hall et al., 2009; Xu et al., 2014; Reese et al., 2016; Epp Schmidt et al., 2017; Lehtimäki et al., 2017).

In our comparisons of beta diversity, composition of bacterial communities grouped by level of urbanization. Composition differed at the site level as well. This is likely because 31 genera were unique to certain levels of urbanization with 24 genera (42% of total genera) unique to a single site. Even though three cosmopolitan genera dominated most communities, sites differed in which OTU within these genera were present. This variation we see in OTU-specific composition could partially be explained by dispersal limitation or neutral processes, at least across small geographic distances.

Our finding that composition varies with land use is in line with our hypotheses: we saw the most variation between individual salamander bacterial communities at exurban sites (Figure 4), and more similarity at urban and suburban sites. Further, when we compare the abundance of the 11 genera found at all three levels of urbanization, urban sites had on average 5–12% more cosmopolitan isolates (a total of 78% of urban isolates) than suburban and exurban sites. In these cases, regional variation is likely driven by local environmental conditions associated with urbanization (i.e., changes in nutrient concentrations, habitat fragmentation, heavy metal deposition, etc.) that overwhelm stochastic factors to select for particularly resilient specialist OTUs as well as cosmopolitan generalists.

This larger role of microenvironment versus stochastic factors has repeatedly been seen in soil communities, where soils with similar environmental characteristics have similar microbial communities regardless of geographic distance (Fierer and Jackson, 2006; Lauber et al., 2009; Xu et al., 2014; Lladó et al., 2018; Plassart et al., 2019). Interestingly, we see this same trend in the bacterial communities of the soil-dwelling salamanders sampled in this study. If skin conditions were the primary selective mechanism, we would see no difference in bacterial composition across our gradient. As others have demonstrated, the soil and water environments in which amphibians live in likely function as species pools providing colonists to amphibian hosts (Loudon et al., 2016), and all of the cosmopolitan genera we found on our salamanders are common soil bacteria known to associate with plants or animals. Thus, our results indicate some combination of contemporary environmental variation and lingering historical effects driving distinct microbial biogeography on these salamanders (Martiny et al., 2006; Kraemer and Kassen, 2015). This study serves as an initial investigation into the overall effects of land use associated environmental changes on microbial communities, but further investigation is needed to determine if and how direct land-use associated effects on the soil bacterial reservoir indirectly alter amphibian cutaneous microbiomes.

It is worth noting that we did not find *Janthinobacterium lividum,* a well-known *Bd*-inhibitory bacteria (Brucker et al., 2008), on any of the *Plethodon cinereus* salamanders sampled despite knowledge that strains have been cultured from aquatic and soil environments in the Hudson Valley of New York (O’Brien et al., 2018). Instead, *Stenotrophomonas*, an antifungal plant mutualist (Wolf et al., 2002; Ryan et al., 2009), was found on salamanders at nearly all sites across our sampling gradient. This genus has a number of favorable characteristics for use as a potential native probiotic in degraded habitats: resistance to heavy metals (including lead), high hydrolytic potential, high bacterial growth rate, and the ability to grow in low-nutrient environments (Ryan et al., 2009). All of these have allowed *Stenotrophomonas* sp. to colonize and compete in many different biotypes adding to its value as potential anti-*Bd* isolate for amphibians in our region.

### Functional Redundancy

Despite the compositional differences we saw in bacterial taxonomy across sites, overall *Bd*-inhibitory ability was similar among urban, suburban, and exurban bacterial communities but varied among individuals. Each site possessed at least one bacterial OTU capable of high *Bd*-inhibition. Given this, *Bd*-inhibition seems to be a functionally redundant characteristic across the gradient, despite differences in the environment and OTU composition. This pattern is not surprising given that naturally occurring bacteria regularly interact and compete with other microbial taxa, including fungi, for space and resources. Whether inhibitory metabolites are *Bd*-specific, fungal-specific, or generally anti-microbial is still undetermined for the majority of bacterial strains isolated from amphibian skin in this and other studies and we encourage continued efforts to characterize the amphibian metabolome to identify the mechanisms responsible for anti-*Bd* function (Woodhams et al., 2014; Belden et al., 2015; Walke et al., 2015).

Despite functional redundancy of *Bd*-inhibition across the gradient, it does not appear to be a trait associated with phylogenetic relatedness among bacteria (Figure 3). We found variation in inhibition across isolates at the phylum, genus, and OTU levels. For example, in comparing isolates from two different individuals with bacterial communities composed of the same genera, we found *Bacillus*, *Pseudomonas*, and *Sphingobacterium* to vary from 8, 9, and 67% *Bd*-inhibition at PB (urban) to 61, 44, and 16% at F1 (exurban). Given this, it might be better for researchers to focus on maintaining functional diversity rather than taxonomic diversity in relation to the conservation of amphibian microbiomes. Overall function of the community considers distribution, abundance, and the products of fluctuating interactions between species in the community (Holling, 2001; Alberti, 2005). As shown in this study, taxonomic differences are poor predictors of community functional differences and presence of particular isolates on amphibians in separate locations does not equate to similarities in function.

### Spatial and Temporal Variation Within Genera and Species

Contrary to our hypothesis, suburban sites associated with medium levels of environmental change appeared to have the largest proportion of inhibitory isolates with 41%, followed by exurban (34%) and urban (30%) sites. In addition, only 32% of all isolates were inhibitory, which suggests that the majority of the salamander microbiomes cultured in this study show little to no effect on *Bd* growth *in vitro*. While inhibition did not significantly differ by level of urbanization, there were site-level differences (Figure 5B). Significant differences among our sites were driven by one suburban site, CC, which consisted of isolates that were on average more inhibitory than all other sites. Differences in *Bd* inhibition among sites could be due to environmental characteristics, absence of particular isolates, changes in overall community composition, or isolate-level trait differences. Often times, these local factors are not reported or considered in other studies using the Anti-fungal Isolates Database to determine community function from high throughput 16S rRNA sequencing. As mentioned before and reiterated by our results, the 16S rRNA gene alone is not a reliable indicator of *Bd*-inhibitory function. Thus, averaging the results of globally distributed functional assays likely blurs the fine-scale differences in microbiome function in different regions and can lead researchers to over- or underestimate microbiome function when not complemented by functional assays from local isolates (Mollet et al., 1997; Fukushima et al., 2002; Hilario et al., 2004; Janda and Abbott, 2007; Becker et al., 2015).

We found a high level of anti-*Bd* functional variation observed across exurban salamander bacterial communities, which was not in line with our initial hypotheses. Our exurban sampling locations (F1, F2, and HH) consist of well-connected hardwood forests and streamside habitats where salamander dispersal is far less limited than in both our urban and suburban sites. However, habitat connectedness or salamander dispersal may not matter at the microbial level. The variation in exurban isolate’s *Bd*-inhibitory function despite high habitat connectivity is not unexpected given that bacteria can engage in horizontal gene transfer and clonal evolution. Studies have shown that multiple clone-types can exist and swap in dominance based on abiotic and biotic community factors (Fenchel, 2003; Devevey et al., 2015; Wright and Vetsigian, 2016). We also observed isolate-level trait differences in comparisons of isolates across sites from the two most dominant genera, *Pseudomonas* and *Bacillus* (Figure 3; Table 2). Less than half of the number of isolates from these genera was inhibitory in urban sites as compared to exurban and suburban sites. These results agree with the findings of Becker et al. (2015), in that there can be substantial functional variation within phylogenetically similar bacterial isolates and provide further evidence for the use of native bacteria in developing potential probiotics.

Differences in an individual bacterial isolate’s interactions with the environment and other microbes are also known to alter bacterial metabolite production (Daskin et al., 2014; Wolz et al., 2017; Medina et al., 2017a; Bird et al., 2018). Thus, another consideration is whether *Bd* is present and when is it most active, as changes in microbial community composition have been linked with the onset of *Bd* infection (Pullen et al., 2010; Longo et al., 2015; Clare et al., 2016; Varela et al., 2018). Specifically, *Bd* is more prevalent during seasons of moderate temperatures (between 17 and 25°C) and high precipitation. In our study, we observed significantly larger *Bd*-inhibition in the fall as opposed to the spring, which is different than that found in previous studies (Figure 5C; Pullen et al., 2010, Varela et al., 2018).

Despite marginal difference in precipitation, Spring in the New York region is generally cooler than Fall (April/May mean = 15.2°C, September/October mean = 18.2°C according to the National Weather Service’s New York City records over the last 10 years). Based on these temperatures, Spring may be too cold for *Bd* growth, whereas Fall is within *Bd*’s optimal temperature range increasing bacterial anti-*Bd* metabolite response (~17–23°C; Piotrowski et al., 2004). Additionally, amphibians have been shown to lower intensity of *Bd* infections through over-wintering (Savage et al., 2011; Longo et al., 2015) or raising their own body temperature (Woodhams et al., 2003), which could have confounding effects on their entire microbiome. These temperature differences both in the environment and on the host could also impact which bacteria are present, their physiologies, and their function. Thus, we believe these results warrant further research into seasonal patterns of *Bd*-inhibition at a range of sites.

## Conclusion

Our results indicate omitting functional assays on locally isolated bacteria can lead researchers to over- or underestimate microbiome function with potentially drastic implications for probiotic development or bioaugmentation. While our study of community variation along a relatively small (<65 km) urban land use gradient complements other regional high-throughput taxonomic analyses, further investigation is needed to determine if and how direct land-use associated effects on the soil bacterial reservoir indirectly alter amphibian cutaneous microbiomes. Additionally, our results highlight the need for research into metabolite composition and the genetic mechanisms responsible for their production. These analyses should continue to be performed in both pristine and altered environments to account for the continued habitat degradation occurring alongside the amphibian disease epidemic. In sum, our results provide further evidence for examining the whole microbiome, including micro-differences in species-species interactions and environmental variability, for understanding how it provides disease protection.

## Data Availability Statement

The sequencing data generated for this study can be found in GenBank using accession numbers MN582766-MN582943.

## Ethics Statement

The animal study was reviewed and approved by Fordham University, Institutional Animal Care and Use Committee Protocol #JL-17-01.

## Author Contributions

EB and JL conceived and designed the experiments and wrote the paper. EB and EC performed the experiments and analyzed the data. EB, EC, and JL contributed materials and reagents and reviewed drafts of the paper.

### Conflict of Interest

The authors declare that the research was conducted in the absence of any commercial or financial relationships that could be construed as a potential conflict of interest.
